# Surface Cleanliness Maintenance with Laminar Flow Based on the Characteristics of Laser-Induced Sputtering Particles in High-Power Laser Systems

**DOI:** 10.3390/mi14030598

**Published:** 2023-03-04

**Authors:** Ge Peng, Qiang Gao, Zhe Dong, Lingxi Liang, Jiaxuan Chen, Chengyu Zhu, Peng Zhang, Lihua Lu

**Affiliations:** 1 Center for Precision Engineering, School of Mechatronics Engineering, Harbin Institute of Technology, West Dazhi Street, Harbin 150001, China; 2 Chongqing Research Institute of Harbin Institute of Technology, Chongqing 401135, China; 3 National Key Laboratory of Science and Technology on Tunable Laser, Harbin Institute of Technology, West Dazhi Street, Harbin 150080, China

**Keywords:** laser-induced damage, fused silica, particle, cleanliness, laminar flow, motion behavior, high-power laser systems

## Abstract

In high-power laser systems, the primary cause of contamination of optical components and degradation of spatial cleanliness is laser-induced sputtering of particles. To mitigate this problem, laminar flow is frequently utilized to control the direction and transport of these particles. This study characterizes the properties of laser-induced sputtering particles, including their flying trend, diameter range, and velocity distribution at varying time intervals. A time-resolved imaging method was employed to damage the rear surface of fused silica using a 355 nm Nd: YAG pump laser. The efficacy of laminar flow in controlling these particles was then assessed, with a particular focus on the influence of laminar flow direction, laminar flow velocity, particle flight height, and particle diameter. Our results indicate that the optimal laminar flow velocity for preventing particle invasion is highly dependent on the maximum particle attenuation distance (or safety distance), which can vary by up to two orders of magnitude. Furthermore, a laminar flow velocity of 0.5 m/s can effectively prevent particle sedimentation. Future research will aim to optimize laminar flow systems based on these findings to achieve high surface cleanliness in high-power laser systems with minimal energy consumption.

## 1. Introduction

In December 2022, scientists at the National Ignition Facility (NIF) [1] achieved a breakthrough by producing 3.15 MJ of energy from an input of 2.05 MJ using laser-induced inertial confinement fusion. This achievement opens up the possibility of obtaining green energy from laser-powered facilities in the future. However, there are substantial challenges that cannot be readily resolved through conventional techniques, for example, high consumptions of (1) optical materials (e.g., fused silica [2] and KDP [3]), (2) ancillary energy [4], and (3) human labor [5]. Similarly, these consumptions are also mandatory in other national laser facilities, including the Laser Mégajoule facility (LMJ) [6] and Shenguang (SG) series [7]. One critical aspect of these consumptions is the usage of ancillary energy, which is mainly required to maintain a high standard of cleanliness in the workspace and on the surface of optical components [8]. This is because laser-induced pollutants present an unavoidable risk [9,10,11].

There are various ways to mitigate the threat, such as by performing offline cleaning of all optical components before target shooting. This includes techniques such as plasma blowing [12], wet washing with detergents [13], ultrasonic [14] or high-pressure spray [15], resonant laser detachment [16], laser ice jet [17], and dry wiping [18], etc. Moreover, online protection using non-contact methods such as applications of electrostatic fields [19] and airflow [20] have become increasingly popular in recent times. For instance, the latest SG series features a dual dynamic airflow protection system that effectively removes particles [21]. Furthermore, advancements in the production and processing of optical [22,23] components have increased their damage threshold, thus reducing the number of laser-induced particles and minimizing particulate threats.

Although the energy consumption is high, there is a consensus that laminar airflow is the most practical online method to maintain long-term cleanliness inside high-power laser systems, particularly for preventing the regeneration of pollutants such as laser-induced particles. When no pump beam is loaded, laminar flow acts as a constant propulsion, entraining particles out of the laser facility. In the SG-III experiment, researchers [24] determined that a minimum inlet velocity of 0.2 m/s should be applied to achieve a continuous flow loop, resulting in a Class 100 cleanliness level near the surface of the optics. Further studies by Zhu et al. [25] divided the laminar flow loop into two rounds and tested it online, revealing a significant improvement in particle elimination and creating a more stable environment in terms of temperature, humidity, and pressure within the final optics assembly. Wang et al. [26] used laminar flow to evaluate the sedimentation efficiency of suspended particles near upward-facing mirrors. They found that after 10 h of ventilation, the cleanliness level returned to Class 100. In NIF, laminar flow is employed as HEPA-filtered ventilation air within portable clean rooms, which helps mitigate airborne (<10 μm) invasion on the surface of the upward-facing optics [13,27]. In high-speed cases [28,29], laminar flow functions as the front part of the ‘air curtain’ created by the gas knife, effectively removing particles from the surface of the optics. However, despite the high removal efficiency of one-pulse gas knife protection, it can still struggle to prevent the re-deposition of particles due to their weight (mass). The cleanliness maintenance near the slabs (the enclosure of laser systems) has also been studied by researchers who used laminar flow with CFD modeling. For example, in 1ω Laser Amplifiers, Spaeth et al. [30] found that a vertical gas purge at a rate of 30% of the enclosure volume per minute can provide a better than Class 1 environment within 6 min.

Laminar flow is also broadly utilized to sustain cleanliness in various industries, such as biological and microelectronic manufacturing [31,32], food production [33,34], and hospital operating rooms [35,36]. The design of flow channels, such as wall-return [37], celling return [38], and radial [39], contributes to the parallel and perpendicular flow of air, providing different levels of protection. Researchers have investigated the factors that impact laminar flow’s effectiveness in maintaining a clean environment and found that the flow velocity (or air change rate) and particle diameter (gravity) play a decisive role, assuming the layout of the clean room is determined (the flow channel is fixed). Regarding laminar velocity, Shih et al. [40] discovered that particle removal efficiency increases with gas velocity as long as particles can move with the main flow. Thongsri et al. [41] observed that while particle removal efficiency improves gradually with increasing laminar airflow velocity between 0.25 m/s and 0.65 m/s within the protected area, it also leads to increased pollution of the surrounding area. The removal efficiency of particles larger than 0.5 μm (concentration limit: >2,000,000 particles/ft^3^ at 5% coincidence loss) was investigated using laminar flow in a vertical-convection clean room [42]. The results showed that as the input velocity of the laminar flow decreased from 0.25 m/s to 0.19 m/s, the protection capability within sub-zones remained strong. However, the lateral dispersion of laminar flow intensified, leading to an increase in particle sedimentation percentage in neighboring zones. In reference to particle diameter (gravity), Tung et al. [43] conducted numerical studies and determined that laminar flow protection (at an average velocity of 0.35 m/s in a 7.8 m × 7.8 m × 3.2 m clean room) is efficient for aerosol particles with diameters below 1 μm. Further experiments with varied layouts confirmed that the laminar flow at this velocity is ineffective in removing particles larger than 100 μm. Verjus et al. [44] explored the effect of laminar flow on particle movement when encountering small barriers. By introducing the stream function, they calculated the minimum diameter of particles that could escape from the local wake and follow the main flow. In addition, Zhao and Wu [45] carried out experiments within a 3 m × 3 m × 4 m clean room, under conditions of a laminar flow at 0.5 m/s on a wall-return ventilation background, to determine the relationship between particle removal efficiency and gravitational sedimentation (diameter). The results of their investigation indicate that, as the particle’s relaxation time can serve as a proxy for passive contamination, particle diameter plays a pivotal role in ensuring the cleanliness of the environment.

Despite the critical significance of laminar flow in maintaining cleanliness, current research is primarily focused on particle removal efficiency as a function of airflow velocity and particle diameter, and the mechanism behind it is yet to be thoroughly studied. In high-power laser systems, focusing solely on particle removal efficiency is inadequate as it will likely result in excessive energy wastage (e.g., the pursuit of velocity improvement). To address this issue, it is essential to comprehend the distinctive characteristics of laser-induced particles of different diameters to build connections with laminar flow, and finally facilitate a modular, clean design (like different line-replaceable units installed in the laser system [46]). However, there is limited knowledge of the transient behaviors of laser-induced particles in aerodynamics. (e.g., how to ascertain a particle’s initial speed along the transient timelines), and the lack of accurate models to predict particle long-range movement, impede further research in this area. On the other hand, in terms of the purge pattern, despite some studies demonstrating that non-unidirectional laminar flow can effectively entrain particles in high-power laser facilities (e.g., laminar flow background and high-speed gas knife can maintain cleanliness for upward-facing mirrors [13,26,27]), there is a dearth of research on the relationship between the protection mechanisms of laminar flow and the aerodynamic properties of aerosol particles in different directions (including gravitational). Take ref. [43] as an example. Although it has been suggested that maintaining cleanliness is related to particle gravity and terminal velocity, the decelerating mechanisms in the longitudinal direction remain largely unclear. In addition, while some studies have incorporated aerodynamic parameters in different directions, the particle source is independent. For example, the particle source within the aerodynamic models created by Zhao and Wu [45] is preset and disconnected from the transient characteristics of the pollutant.

This work overcomes the aforementioned limitations by associating the characteristics of laser-induced sputtering particles with the protection mechanism of laminar flow to examine how laminar flow velocity and particle diameter affect the removal efficiency. An aerodynamic model based on the Reynolds number is used to quantitatively determine the motion of the particles in the air in both the longitudinal and gravitational directions. First, we captured the transient characteristics of the laser-induced particles using a time-resolved system, where a 355 nm Nd: YAG pump laser was used to damage the rear surface of fused silica. The particle diameter range and the longitudinal velocity at different time delays are then determined from the transient data. Second, the speed of particles at 13,000 ns is estimated through a decay function that is fitted with the velocities at different time delays. This estimation is thereafter utilized as the input for the aerodynamic model to predict the long-range movement of the particles, including the maximum distance they will attenuate. This process refers to some interesting aerodynamic phenomena of particles in various directions of motion. Finally, the impact of different laminar flow velocities (particle flying heights) on the removal efficiency of laminar flow is compared. The results of this work provide guidelines for using laminar flow to maintain high surface cleanliness in high-power laser systems.

## 2. Experimental Design

**Figure 1:**
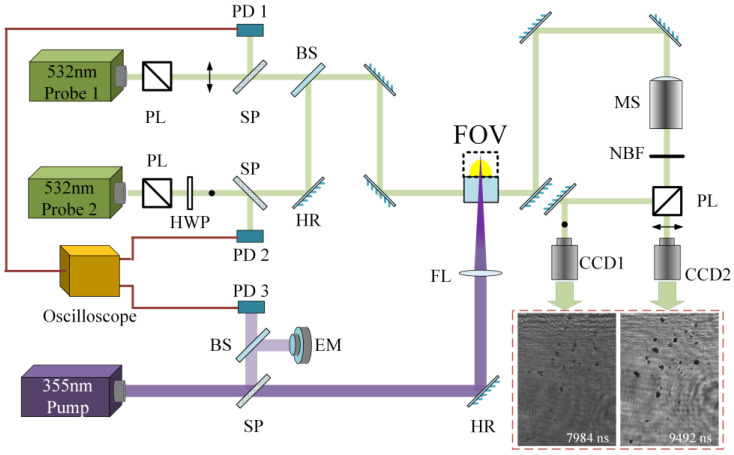
Schematic of time-resolved imaging for capturing transient particles after a laser-induced breakdown event. PD: photon detector, PL: polarized lens, SP: sampler, HWP: half-wave plate, BS: beam splitter, FL: focus lens, NBF: narrowband filter, MS: microscope, HR: high reflector, EM: energy meter, FOV: field of view.

Figure 1 depicts a time-resolved microscope system capable of capturing the initial characteristics of particles produced by a laser-induced breakdown event. The system has a temporal resolution ranging from tens of nanoseconds to over ten microseconds, with a maximum spatial resolution of 0.72 μm/pix and a field of view of 2.4 mm × 3.4 mm. The pump laser used was an Nd: YAG laser operating at 355 nm with a pulse duration of approximately 12 ns (measured as the full width at half maximum, FWHM). This laser was focused on the rear surface of a target (JGS1 fused silica with a surface area of 50 mm × 50 mm and a thickness of 5 mm) using a focus lens (150 mm focal length). By adjusting the pump’s power, a Gaussian laser spot with a diameter of about 0.6 mm was created on the surface, causing damage at fluences ranging from an average of 18 J/cm^2^ to 79 J/cm^2^, with an estimated probability of 85%. An Nd: YAG probe laser (Probe 1) at 532 nm with a pulse duration of approximately 9 ns (as measured by the FWHM) was used to capture transient images with a constant timeline during a single breakdown event. This probe laser was synchronized with the pump laser and was controlled by a DG645 digital delay generator to adjust the sequence of time delays. The timing error of the probe laser was less than 10%, according to the pump. The images were captured by a CCD camera with a field of view perpendicular to the target surface. To obtain transient characteristics at a specific time delay, a second probe laser (Probe 2, with the same parameters) was used simultaneously at an adjacent time delay in an orthometric direction. The time interval between Probe 1 and Probe 2 can be adjusted based on the timing of material ejection after the laser-induced breakdown.

In this experiment, each portion of the pristine surfaces of the target was subjected to a solitary pulse, with a minimum interval of 2.5 mm separating each other. Each laser fluence was repeated thrice. To maintain a clean level of at least 100 degrees (monitored by two perpendicular particle counters, Beckman Coulter’s MET ONE 6000, within a volume of 0.5 m × 0.5 m × 0.5 m) before each shot, a fan filter unit was utilized to create vertically laminar flows with an average speed of 0.42 m/s (monitored by an anemometer, Model 9565 series, VELOLICALC).

## 3. Particle Flight Equations

**Figure 2:**
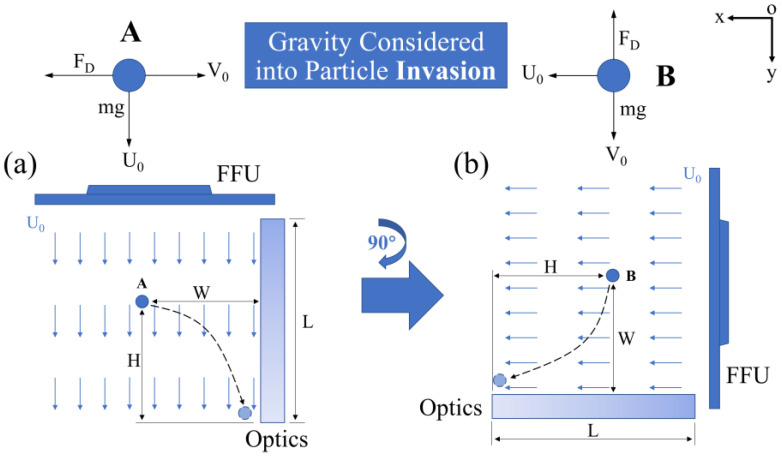
Two methods preventing particulate invasion by laminar flow, (**a**) avoid direct impact for particles with a longitudinal velocity of *V*_0_; (**b**) sedimentation control after *V*_0_ decreases to 0.

In high-power laser systems, optical components are typically arranged either vertically (y-direction), as depicted in Figure 2a, or longitudinally (x-direction), as shown in Figure 2b. These two distinct orientations are susceptible to particle invasion in the form of (1) a direct hit with an initial longitudinal velocity of *V*_0_ (statistically obtained after a laser-induced breakdown event) and (2) free deposition vertically when *V*_0_ decreases to 0 after a long-range movement. During this period, the laminar flow maintains a constant velocity of *U*_0_ parallel to the surface of the components. To prevent particle invasion, the particle flight Equation in the x and y directions for scenario (1) must meet the following criteria:

(1) $$U_{0}\cdot t+G(t)\geq H$$

(2) L(t)≤W

where, *t* is the elapsed time during particle flight, *G*(*t*) represents the translation distance caused by gravitational forces, and *L*(*t*) is the translation distance in the x-direction. It is important to note that *G*(*t*) is dependent on a special time, *t_t_*, at which the particle reaches its terminal velocity, *v_t_*, under the influence of gravity (in the y-direction). For *t* > *t_t_*, *G*(*t*) is comprised of both accelerated and uniform terms, which can be expressed as:

(3) $$G(t)=\int_{0}^{t_{t}}v(t)dt+v_{t}(t-t_{t})$$

otherwise, when *t* < *t_t_*, the equation becomes:

(4) $$G(t)=\int_{0}^{t}v(t)dt$$

where, *v*(*t*) ≤ *v_t_*.

*G*(*t*) and *L*(*t*) are Equation that describe the motion of a single spherical particle in the presence or absence of gravity, and they are derived from the governing equation of particle motion [44]:

(5) $$m\frac{dv}{dt}=mg-F_{D}$$

where, *m* is the particle mass, *v* is the transient velocity of the particle, *g* is the acceleration due to gravity, and *F_D_* represents the drag force experienced by the particle in the air. By substituting the transient velocity *v* with Reynolds number *Re*, Equation (5) can be reformulated:

(6) $$\frac{dRe}{dt}=\frac{1}{24\tau }(\zeta -C_{D}Re^{2})$$

where *τ* is the relaxation time of the particle, which is dependent on *ρ_p_*, *d_p_*, and *μ_g_*. The constant *ζ* incorporates all parameters in *τ*, as well as the *g* and *ρ_g_*. The drag coefficient of the particle *C_D_*, which is a function of the *Re*, represents the resistance of the particle in an incompressible flow. In this study, the *C_D_* is simplified into three regimes [47]: the Stokes regime (*C_D_* = 24/*Re*, *Re* < 1), the Allen regime (*C_D_* = 10.6/*Re*^1/2^, 1 < *Re* < 500), and the Newton regime (*C_D_* ≈ 0.44, 500 < *Re* < 2 × 10^5^). By integrating Equation (6), the *Re*-dependent functions of the particle’s translational distances in the y-direction (*G*(*t*)) and x-direction (*L*(*t*), with *ζ* = 0), and elapsed time during particle flight (*t*) can be expressed as:

(7) $$G(t)=\frac{4}{3}\frac{\rho_{p}}{\rho_{g}}d_{p}\int_{Re_{0}}^{Re}\frac{Re}{\xi -C_{D}Re^{2}}dRe$$

(8) $$t=24\tau \int_{Re_{0}}^{Re}\frac{1}{\xi -C_{D}Re^{2}}dRe$$

where, *Re*_0_ is the initial Reynolds number at time *t* = 0, and *Re* corresponds to the transient velocity *v*(*t*) that is less than *v_t_*. The terminal velocity *v_t_* is determined when the derivative of *Re* concerning time, d*Re*/d*t*, equals 0. In this case, since *C_D_* belongs to the Stokes regime, *v_t_* is defined as:

(9) $$v_{t}=\frac{(\rho_{p}-\rho_{g})g}{18\mu_{g}}d_{p}^{2}$$

For scenario (2), laminar flow longitudinally entrains particles and prevents them from landing on the surface of the optics with an edge length of *L*. As laser-generated particles are primarily aerosols with diameters smaller than 50 μm [9,11], they are easily influenced by the flow, and their inertial effect along the x-direction can be disregarded. Additionally, the velocity of the laminar flow inside high-power laser systems is typically less than several meters per second [13,21]. The air convection along the z-direction (perpendicular to the xy plane) can also be disregarded. Based on these assumptions, the longitudinal trajectory *x* of a particle is described as:

(10) $$\frac{dx}{dt}=u=U_{0}$$

where *u* is the longitudinal velocity.

The vertical trajectory of a particle is solely regulated by *G(t)*, as expressed in Equation (7). To showcase a similar equation to Equation (10), incorporating the vertical velocity, *v*, the balance condition within the Stokes regime, where ζ = 24 *Re_t_* (attaining the terminal speed, *v_t_*), and the ratios *Re*_0_/*Re_t_* = *V*_0_/*v_t_*, *Re*/*Re_t_* = *v*/*v_t_* and *τ*, are introduced into Equation (8). Thus, Equation (7) can be re-written as follows:

(11) $$\frac{dy}{dt}=v-v_{t}(1-e^{-t/\tau })$$

For fused silica particles with a diameter ranging from 1 to 50 μm and a density of 2200 kg/m^3^, moving at 20 °C and 1 atm (where *μ_g_* = 1.816 × 10^−5^ Pa∙s), the *τ* varies from 6.73 × 10^−6^ s to 1.68 × 10^−2^ s, which are noticeably smaller compared with their stasis time above the surface of the optics. As a result, the term *e*^−*t*^/*^τ^* can be disregarded. In a 2D plane model, the particle velocities *u* and *v* can be represented by differentiating the stream function *ψ* (x, y) in opposite directions [44]. By incorporating these into Equations (10) and (11), and integrating over the edge length *L*, the variation of *ψ*, which denotes the particle volume flow rate per unit height (in the y direction), can be calculated as follows:

(12) $$\Delta \psi =\psi_{L}-\psi_{0}=v_{t}L$$

where the subscript 0 or *L* refers to the translational distance in the y-direction. As per the definition of streamlines, which do not cross one another, *ψ*_0_ ≡ 0. In this case, *ψ_L_* determines the flow volume of particles descending onto the surface. Furthermore, at the front edge of the optics, if the total volume of particles across a vertical line with height *W* above the surface is designated as *Q*, the protection efficiency of laminar flow (particle removal efficiency), *η*, which represents the percentage of particles not sinking onto the surface of the optics, is defined as follows:

(13) $$\eta =1-\frac{\psi_{L}}{Q}=1-\frac{v_{t}L}{\int_{0}^{W}udy}=1-\frac{v_{t}L}{U_{0}W}$$

## 4. Results and Discussion

**Figure 3:**
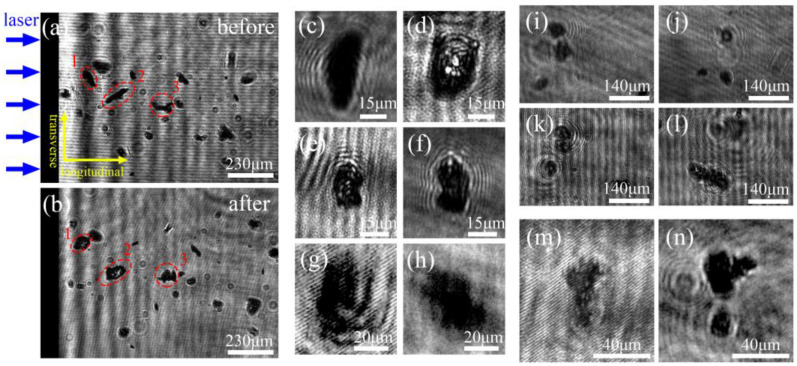
Transient characteristics of laser-induced particles acquired by comparing two images at different time delays at the laser fluence of 79 J/cm^2^. (**a**,**b**), (**i**,**j**), and (**k**,**l**) at 3982 ns to 5504 ns, (**c**,**d**), and (**e**,**f**) at 3985 ns to 5997 ns, (**g**,**h**), and (**m**,**n**) at 5987 ns to 7495 ns.

The transient behavior of particles is illustrated in Figure 3 by comparing two consecutive images taken at adjacent probe time intervals. The probe intervals for (a)–(b), (i)–(j), and (k)–(l) range from 3982 ns to 5997 ns; (c)–(d) and (e)–(f) range from 3985 ns to 5504 ns; (g)–(h) and (m)–(n) range from 5987 ns to 7495 ns. It is evident that there are two types of translational shifts and three types of rotational shifts in the longitudinal (x) and transverse (y) directions: (i)–(j) depict the “catch-up” characteristics of three particles with changes in the x direction, while (k)–(l) show a similar occurrence with changes in the y direction. Particle 3 in (a)–(b) rotates along the x-axis, while the particles in (c)–(d) and (e)–(f) rotate along the y-axis and z-axis (perpendicular to the xy plane), respectively. Although the translational shift in the z direction is not visually detectable, particle motion is present. On the one hand, the particle motion follows the shock wave front that propagates spherically in a power-law medium after the laser-induced explosion [48]. On the other hand, the laser-induced damage site on fused silica is crater-shaped and contains circumferential and radial cracks, suggesting anisotropic and three-dimensional material ejection [49]. Thus, transient particle motion encompasses six degrees of freedom.

The six degrees of freedom present in the transient characteristics of particles contain information on their movement, both in terms of translational and rotational movements. Out of these, only the longitudinal velocity of *V*_0_ derived from the translational distance in the x-direction contributes to the particle’s direct invasion (as displayed in Figure 2a). The remaining translational distances in the y and z directions, which arise due to factors such as energy deposition [11], temporal air acceleration caused by pressure gradient differences [50], and Basset history [51], only change the relative positions of particles before they reach the surface. Additionally, rotational movements of particles in all directions during flight, which result in changes in Magnus and Saffman lift forces [52,53], only further modify the translational distances in the y and z-directions. By subtracting the translational distance from the rear surface of the sample in each image (i.e., *S*_2_ − *S*_1_) and dividing by the interval of adjacent time delays (i.e., Δ*t*), the *V*_0_ can be calculated as:

(14) $$V_{0}=\frac{S_{2}-S_{1}}{\Delta t}$$

**Figure 4:**
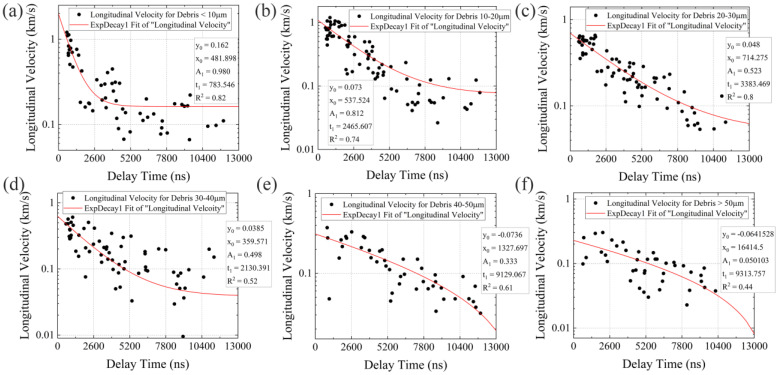
Longitudinal velocity *V*_0_ of particles with different diameters vs. probe time delays ranging from 500 to 12,000 ns, where (**a**) represents particles with diameters <10 μm, (**b**) 10–20 μm, (**c**) 20–30 μm, (**d**) 30–40 μm, (**e**) 40–50 μm, and (**f**) >50 μm, red curves represent fit decay functions.

Equation (14) used to determine the longitudinal velocity of the particles (*V*_0_) assumes low energy loss and neglects particle detachment during flight, as evidenced by Figure 3m,n. This equation offers a more accurate determination of *V*_0_, as it eliminates the error between the sputtering time and probe delay [10]. The results of *V*_0_ as a function of time delay, ranging from 500 ns to 12,000 ns, for various particles are shown in Figure 4, with the laser fluence set at 79 J/cm^2^. Particles with diameters less than 20 μm and velocities greater than 1.2 km/s are predominantly observed at early time delays, but their velocities drop to around 100 m/s at later time delays. Particles with diameters between 20 and 40 μm exhibit a similar scattering pattern, with a maximum velocity at early time delays of approximately 0.65 km/s. Their velocity continues to decrease at later time delays, though not significantly. The scattering pattern of particles larger than 40 μm is more varied, with most appearing at the middle and later time delays and having velocities below 100 m/s. Additionally, there is a decrease in their numbers, particularly those observed at early time delays.

To present the trends of particle velocity *V*_0_ within the probe time delays and obtain precise estimates before their impact on the surface of the optics, a set of fit functions based on the exponential decay archetype were plotted to encapsulate the scattered data points shown in Figure 4 in the following format:

(15) $$f(x)=y_{0}+A_{1}\cdot \exp(-(x-x_{0})/t_{1})$$

where *y*_0_, *A*_1_, *x*_0_, and *t*_1_ are constants that are not dependent on any physical variables. There are two trends in the fitted curves based on *y_0_*: a gradual slowdown with time delays Figure 4a–d, *y*_0_ > 0, and a decline followed by a sharp drop (Figure 4e,f, *y*_0_ < 0). The former trend is observed for particles with diameters <40 μm, while the latter is for those with diameters >40 μm. This suggests that smaller particles undergo a much quicker decelerating process in their early launch stages. For example, in the case of particles with a diameter <10 μm, the sharp deceleration process only lasts for approximately 2600 ns, which is consistent with some transient observations [9,54]. This can be attributed to backward propulsion caused by changes in air density (similar to lateral translations) and the electrostatic force from exposure to high fluence irradiation [55]. Additionally, from an aerodynamics perspective, the sharp drop in velocity of the fitted curve approaches the analytical results solved by Equation (5), suggesting the outline of these small particles is similar to a sphere. Conversely, there are no significant velocity changes for larger particles during their early launch stages, possibly due to a lesser influence from the factors discussed above. This is possible because larger particles are generated through material pulverization caused by the release of compressive stress [9] instead of thermal ablation (for small particles), and the ejection mechanism complies with failure laws and disregards laser-induced disturbance. A majority of these large particles have flat outlines and thin thicknesses, so they are likely to encounter increasing periodic air resistance (with the flat side changing orientation during flight) after the early launch stages. As a result, their velocity drops sharply with limited translational distance in the x-direction. The detailed mechanism of deceleration for large particles has been discussed in refs. [11,19] and will not be repeated in this work.

**Figure 5:**
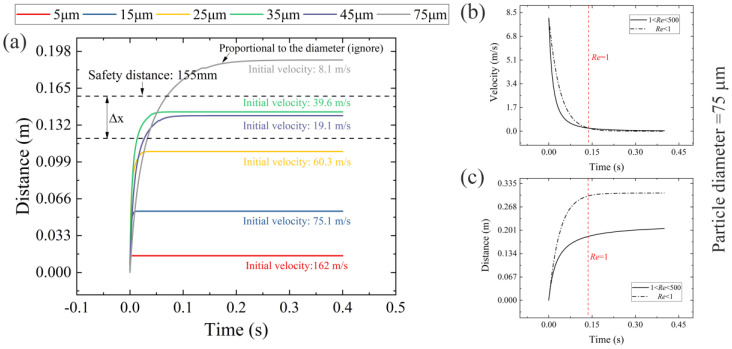
(**a**) Particle’s maximum attenuation distance vs. time calculated by Equation (5) using velocities fitted at 13,000 ns; (**b**,**c**) are plots of particle velocity and distance vs. time governed by Allen and Stokes regime, respectively, where particle diameter range >50 μm.

The fit functions accurately depict the transient characteristics of laser-induced particles, as they have been evaluated over a wide range of diameters and at preset time delays ranging from 500 ns to 12,000 ns. These functions are then extrapolated at 13,000 ns to determine the initial velocity (*V*_0_) of the particles before they invade the surface of the optics. The *V*_0_ and corresponding Reynolds numbers for different diameter ranges (take the median) are presented in Table 1. The total distance traveled by the particles away from the target’s rear surface, known as the attenuation distance (*L*(*t*)), is divided into two parts. The first part is governed by the fit functions, which consider most factors related to laser-induced ablation, radiation, fracture, and short-term aerodynamic instability. The second part, in contrast, is governed by the stable surrounding air and is described by Equation (5). As the first part only ranges from 1.7 to 3.8 mm ((integrate fit functions between 0 ns and 13,000 ns)), which is a small fraction of the total translational distance in the x-direction (beyond 72 mm [11]), the second part primarily influences the attenuation distance. Figure 5a displays the attenuation distances of the particles over time, as calculated using the initial velocities obtained from the fit functions at 13,000 ns and Equation (5).

In Figure 5a, it is evident that there is a significant rise in the particle attenuation distance in a short period, followed by a steady increase until reaching its maximum. This is due to a change in the drag coefficient (*C_D_*) when the Reynolds number (*Re*) drops to 1. There are only two regimes, Allen (with 1 < *Re* < 500) and Stokes (with *Re* < 1), that govern the change in *C_D_*, in contrast to the situation when particles have hypervelocity (e.g., at the start of particle sputtering, where *Re* exceeds 500 [10]). Since the *Re* > 1 regime accounts for the majority of the increase in the distance during particle deceleration and the trend in the *Re* < 1 regime is similar to that in the Stokes regime (i.e., there is no substantial decline in velocity or increase in distance, as seen in Figure 5b,c with a particle diameter of 75 μm as an example), the Allen regime is a suitable choice for predicting the maximum attenuation distance throughout the entire timeline. This valuable discovery allows us to quickly pre-calculate the safety distance (beyond which any optical component downstream remains self-cleaning without the use of laminar flows) without the need for precise modeling (e.g., a CFD-DEM coupling method based on the variation of drag and lift coefficients [47]) or experimentation (e.g., using a particle counter [42]), once we have obtained *V*_0_. It was also found that the predicted maximum attenuation distance will increase further by substituting *C_D_* in the Stokes regime, and the increase is greater for larger particles (as shown in Figure 5c). However, for the long-range movement of particles under the preset laser fluences and incident spot sizes, this prediction appears to deviate more from reality [11]. Additionally, we discovered an interesting result: the time it takes for particles to move in the x-direction from *V*_0_ (corresponding Reynolds number) to *Re* = 1 is close to the time they reach their terminal velocity, as shown in Table 2. This connection between the two directional movements of particles in one graph in terms of curve variation helps us calculate the vertical distance with the proper equation. For example, given a specified longitudinal distance with *Re* > 1 (the moving time, *t*, can then be determined), particles must be vertically accelerated, and the vertical distance is calculated using Equation (4). As a result, the remaining distance determined by the laminar flow can be established, as can the velocity, *U*_0_. The choice of *U*_0_ based on the predefined attenuation distance will be discussed in the next section. Since Figure 3a can be substituted for the curves controlled by the Allen regime, its properties are inherited as well.

As discussed above, the determination of the *U*_0_ of the laminar flow involves obtaining the safety distance, which represents the predicted maximum attenuation distance of particles of various diameters. As shown in Figure 5a, this parameter is represented by a straight line perpendicular to the y-axis. However, the prediction deviates significantly for particle diameters larger than 40 μm, with increasing deviation as the diameter increases (refer to Figure 5b and Figure 4f). Therefore, particle diameters larger than 50 μm are excluded from this discussion. A safety distance of 155 mm, slightly larger than the predicted maximum attenuation distance of remaining particles, is assumed. By subtracting Δ*x* from this safety distance (expressed as *λ* = Δ*x*/S), the lowest *U*_0_ and maximum *H* at which the optical components downstream can remain clean can be obtained. The values of *U*_0_ and *H* without considering particle gravity are marked as *U_0_′* and *H′*, respectively. Likewise, we define *α* = /*U*_0_, and *β* = /*H* to make comparisons. For large aperture optical components, their chord length is generally greater than 360 mm [24], and they are likely to encounter particulate threats within this range. Thus, we use this value as their initial height *H_0_* (for determining the lowest *U*_0_). It should be noted that there are two cases for (S-Δ*x*), with particle motion governed by the Allen regime (*Re* > 1) or the Stokes regime (*Re* < 1), resulting in varying *U*_0_. Table 3 shows the lowest *U*_0_ of the laminar flow applied at different *λ* values.

In Table 3, the first five rows show the case where (S-Δ*x*) is governed by the Allen regime (*Re* > 1). For *λ* = 25.8% (S-Δ*x* = 115.01 mm), particles with diameters smaller than 30 μm do not move far enough that applying laminar flow is unnecessary. However, for particles with diameters between 30 and 40 μm, the lowest required *U*_0_ is 30.86 m/s. Note that the maximum attenuation distance for these particles is 143.8 mm, implying that the high velocity of the laminar flow only reduces their x-direction translation distance by about 28 mm. As particle diameter increases to 40–50 μm, the lowest *U*_0_ decreases to 16.03 m/s, which is half the velocity required for particles within 30–40 μm. This result suggests that the increase in particle mass decreases the translation distance in the x-direction, leading to a decrease in the lowest velocity of laminar flow. However, for particles with equal mass, gravity has a negligible effect as *α* approaches 1. For *λ* = 53.2% (S-Δ*x* = 72.54 mm), (S-Δ*x*) is equal to the maximum attenuation distance of the particles within 20–30 μm, and the lowest required *U*_0_ is 74.52 m/s. For particles within 30–40 μm and 40–50 μm, the lowest *U*_0_ is 75.93 m/s and 40.41 m/s, respectively. As Δ*x* increases, *U*_0_ increases by more than double, and the mass effect still matters (although it weakens for particles within 20–30 μm). Since *α* still approaches 1, the impact of gravity is also diminished. In conclusion, the lowest *U*_0_ should be selected from the higher value, i.e., 30.86 m/s for *λ* = 25.8% and 75.93 m/s for *λ* = 53.2%.

The remaining rows in Table 3 show the case where (S-Δ*x*) is governed by the Stokes regime (*Re* < 1). In this case, (S-Δ*x*) is positioned near the maximum attenuation distance of particles with a particular diameter range. In contrast, for others, it may be governed by the Allen regime (as calculated before) or surpass the maximum attenuation distance. Therefore, we only compare the lowest *U*_0_ for particles of different diameters. For particles within 20–30 μm (*λ* = 30%), the lowest *U*_0_ is 8.2 m/s, while for larger particles, it decreases to 3.96 m/s (30–40 μm) and 2.42 m/s (40–50 μm). This result suggests that the particle mass (not gravity, due to *α* approaching 1) also reduces the lowest value of *U*_0_. Furthermore, it is observed that the lowest *U*_0_ decreases by an order of magnitude and reaches a value commonly used in ventilation systems of high-power laser facilities [21,25,26]. However, locating (S-Δ*x*) at the Allen regime significantly increases the lowest *U*_0_, as seen with *λ* = 30% (near the maximum attenuation distance for particles within 20–30 μm). To avoid all particle threats, including those within 30–40 μm and 40–50 μm, the lowest *U*_0_ has to be greater than 30.86 m/s (for particles within 30–40 μm). Therefore, the optimal location for (S-Δ*x*) is where only the Stokes regime operates, i.e., the curve closest to the safety distance S. The lowest *U*_0_, in this case, is chosen as 3.96 m/s (for particles within 30–40 μm).

Table 4 displays the maximum height (*H*) that particles can reach before reaching the optics for different *λ* values, with the laminar flow velocity (*U*_0_) fixed at 0.1 m/s, 0.5 m/s, and 1 m/s. Similar to before, *λ* = 25.8% and 53.2% fall under the Allen regime, while the rest fall under the Stokes regime.

For the Allen regime (*Re* > 1), it is observed that the heavier the particle, the higher its maximum *H* in a laminar flow with a fixed *U*_0_. The effect of gravity on the maximum *H* is also significant, as demonstrated by the decrease in *β* from 72.68% to 59.54% when *λ* = 25.8% and *U*_0_ = 0.1 m/s. However, as *U*_0_ increases, the influence of gravity weakens, particularly at *U*_0_ = 1 m/s (*β* will surpass 90%), which suggests a velocity threshold for laminar flow design, i.e., beyond a velocity of 1 m/s, particle motion in the gravitational direction is primarily controlled by the laminar flow, and particle gravity affects less. As *U*_0_ increases, the maximum *H* increases simultaneously, while the behavior is less pronounced for larger particles. This is because larger particles have a more challenging time following the background flow, as defined by the Stokes number. Since the maximum *H* values are in the range of 10^−4^ m to 10^−2^ m (which is significantly shorter than the optics length) and laser-induced damage on the surface of the optics is rarely at the edge [7], it suggests that the laminar flow with preset velocities is unlikely to prevent particle invasion.

In addition to the above findings, it is noted that in the case of Stokes-governed flow (*Re* < 1), there is a further increase in the maximum *H*, with average values reaching 10^−2^ m orders of magnitude. For particles within 40–50 μm, the maximum *H* reaches 159 mm, nearly half the optics’ length. This suggests that laminar flow, with velocities ranging from 0.1 m/s to 1 m/s, demonstrates an enhanced capability in avoiding particle invasion for a particular particle diameter range when applied close to the particle’s maximum attenuation distance. More specifically, all particle invasions can be successfully prevented if applied near the safety distance.

**Figure 6:**
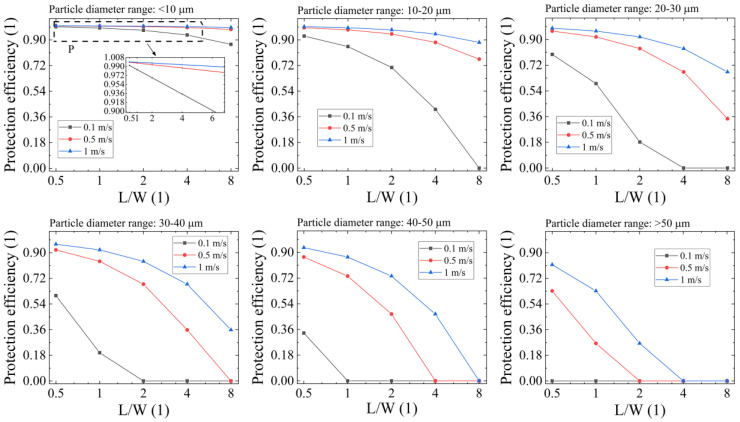
Protection efficiency of laminar flow (*η*) vs. particle height ratio (*L*/*W*) in preventing particle invasion during free-sinking, where particle diameter ranges from <10 μm to >50 μm.

The protection efficiency, *η*, of the laminar flow in preventing particle invasion during free-sinking (as depicted in Figure 2b, scenario 2) is a function of the laminar velocity *U*_0_, the particle’s initial height *W*, the particle’s terminal velocity *v_t_* (i.e., particle mass), and the optics length *L*, as calculated by Equation (13). For this analysis, *L* is preset to 360 mm, and *U*_0_ is set to 0.1 m/s, 0.5 m/s, and 1 m/s for particles with diameters ranging from <10 μm to >50 μm. The result of *η* vs. *L*/*W* is shown in Figure 6. For particles <10 μm, the *η* calculated by Equation (13) remains high, even if initially ejected at the height of 1/8 of the length of the optic with a low *U*_0_ of 0.1 m/s, reaching a value of 86.9%. However, *η* declines significantly for particles with diameters >50 μm. With *U*_0_ = 0.1 m/s, *η* ≡ 0, indicating that the laminar flow is ineffective in preventing particle invasion even when the particle is ejected at twice the length of the optics. The laminar flow remains ineffective even with *U*_0_ set to 1 m/s when *L*/*W* = 4. Assuming that the laser-induced breakdown occurs at the height of half the optics length (i.e., *L*/*W* = 2), the laminar flow with *U*_0_ = 0.1 m/s is ineffective in preventing particle sedimentation with diameters >20 μm, as *η* < 50%. However, when *U*_0_ is increased to 0.5 m/s, *η* rises to at least 67.9%, except for particles with large diameters (>40 μm). Since larger particles settle immediately at the bottom of the optics after laser-induced damage (not in long-range movement), this result suggests that a typical velocity setting of laminar flow around 0.5 m/s is reasonable for mitigating the threat of sedimented laser-induced particles in high-power laser systems.

## 5. Conclusions

This study focuses on maintaining the surface cleanliness of optical components in high-power laser systems through laminar flow. The investigation analyzes the transient sputtering behavior of laser-induced fused silica particles and aims to determine the best practices for controlling their movement and preventing contamination. The key findings of this study are:

(1) The movement of particles after initial deceleration (13,000 ns) is governed by two regimes (classified by Reynolds numbers), the Allen regime and the Stokes regime, with the Allen regime being the dominant factor.
(2) The time it takes for particles to reach their terminal velocity vertically is approximately the same as the time it takes to transition from their initial velocity (corresponding Reynolds number) to *Re* = 1, but much shorter than the time it takes to transition from *Re* = 1 to *Re* = 0.
(3) To prevent particles from directly invading, a hypervelocity laminar flow is necessary when the interval between optical components is less than the safety distance (particle’s maximum attenuation distance). However, as the interval approaches the safety distance, the laminar flow velocity decreases to several meters per second. Therefore, increasing the interval between optical components closer to the safety distance during application is more cost-effective.
(4) If it is not feasible to reduce the distance within the safety distance, alternative measures, such as using a gas knife, may be considered in the future.
(5) A laminar flow velocity greater than 1 m/s allows the particle’s gravitational motion to be controlled solely by the flow. This observation provides insight into designing flow-based clean systems.
(6) A laminar flow velocity of 0.5 m/s can effectively prevent particle sedimentation.

Overall, this study provides valuable information for optimizing laminar flow systems and achieving high surface cleanliness in high-power laser systems with minimal energy consumption.

## Figures and Tables

**Table 1:** Longitudinal velocity (Reynold number) of particles extrapolated at 13,000 ns.

Particle Diameter (μm)	Velocity Extrapolated at 13,000 ns (m/s)	Reynolds Number (1)
<10 (5)	162	53.93
10–20 (15)	75.1	75.01
20–30 (25)	60.3	100.36
30–40 (35)	39.6	92.27
40–50 (45)	19.1	57.22
>50 (75)	8.1	40.40

**Table 2:** Time to reach (1) particle terminal velocity, and (2) *Re* = 1 in the x-direction (longitudinally).

Particle Diameter (μm)	Time to Reach Terminal Velocity (s)	Time to Reach *Re* = 1 in the x-Direction (s)
<10 (5)	7.77 × 10^−4^	9.62 × 10^−4^
10–20 (15)	7.00 × 10^−3^	6.04 × 10^−3^
20–30 (25)	1.94 × 10^−2^	1.63 × 10^−2^
30–40 (35)	3.81 × 10^−2^	3.34 × 10^−2^
40–50 (45)	6.30 × 10^−2^	5.37 × 10^−2^
>50 (75)	1.75 × 10^−1^	1.45 × 10^−1^

**Table 3:** Recommended lowest *U*_0_ of laminar flow applied at different *λ* (below maximum attenuation distance).

*λ* (1)	Particle Diameter (μm)	Flying Time (s)	Lowest *U*_0_ (m/s)	*α*
25.8%	30–40	0.01165	30.86	99.88%
	40–50	0.02237	16.03	99.58%
53.2%	20–30	0.00483	74.52	99.98%
	30–40	0.00474	75.93	99.97%
	40–50	0.00890	40.41	99.91%
30%	20–30	0.04373	8.20	99.55%
7.2%	30–40	0.08923	3.96	98.18%
9.4%	40–50	0.14140	2.42	95.24%

**Table 4:** Maximum *H* of particles before reaching the optics surface at different *λ* (below maximum attenuation distance).

*λ* (1)	Particle Diameter (μm)	Flying Time (s)	*U*_0_ (m/s)	Maximum *H* (m)	*β* (1)
25.8%	30–40	0.01165	0.1	1.60 × 10^−3^	72.68%
	40–50	0.02237	0.1	3.76 × 10^−3^	59.54%
	30–40	0.01165	0.5	6.26 × 10^−3^	93.01%
	40–50	0.02237	0.5	1.27 × 10^−2^	88.04%
	30–40	0.01165	1	1.21 × 10^−2^	96.38%
	40–50	0.02237	1	2.39 × 10^−2^	93.64%
53.2%	20–30	0.00483	0.1	5.64 × 10^−4^	85.66%
	30–40	0.00474	0.1	5.66 × 10^−4^	83.71%
	40–50	0.00890	0.1	1.21 × 10^−3^	73.66%
	20–30	0.00483	0.5	2.50 × 10^−3^	96.76%
	30–40	0.00474	0.5	2.46 × 10^−3^	96.25%
	40–50	0.00890	0.5	4.77 × 10^−3^	93.33%
	20–30	0.00483	1	4.91 × 10^−3^	98.35%
	30–40	0.00474	1	4.83 × 10^−3^	98.09%
	40–50	0.00890	1	9.22 × 10^−3^	96.55%
30%	20–30	0.04373	0.1	6.00 × 10^−3^	72.85%
			0.5	2.35 × 10^−2^	93.06%
			1	4.54 × 10^−2^	96.41%
7.2%	30–40	0.08923	0.1	1.55 × 10^−2^	57.67%
			0.5	5.12 × 10^−2^	87.20%
			1	9.58 × 10^−2^	93.16%
9.4%	40–50	0.14140	0.1	3.13 × 10^−2^	45.23%
			0.5	8.78 × 10^−2^	80.51%
			1	1.59 × 10^−1^	89.20%

## Data Availability

Data underlying the results presented in this paper is not publicly available at this time but may be obtained from the authors upon reasonable request.
